# Medical teaching beyond graduation: undergraduate study groups

**DOI:** 10.1590/S1516-31802010000500002

**Published:** 2010-09-02

**Affiliations:** I MD, PhD. Associate professor, Department of Cardiopulmonology, Faculdade de Medicina da Universidade de São Paulo (FMUSP), São Paulo, Brazil.; II MD. Postgraduate student in the Discipline of Thoracic and Cardiovascular Surgery, Faculdade de Medicina da Universidade de São Paulo (FMUSP), São Paulo, Brazil.

Continuing with our series of discussion papers on extension activities and their importance for present-day medical training, we will now cover undergraduate study groups within medicine. Although there are different formats for such entities, we can define them as nonprofit student organizations that create opportunities for instructional, scientific, cultural and social activities for their members, always covering a certain field within healthcare, with the aims of achieving learning and development. They are administered by the students themselves, but with guidance from teaching staff.^1^

There is some debate regarding whether this type of activity helps or hinders undergraduate teaching. This is because many students end up leaving aside activities belonging to the medical course in order to dedicate themselves to activities within undergraduate study groups. Another negative point that has been made is that some students regard such activities as a chance to achieve "early specialization". Thus, they dedicate themselves excessively to one field, for example plastic surgery or rheumatology, without taking sufficient interest in other, equally important areas of general training.^2^

However, the positive points that can be made in relation to the activities of undergraduate study groups seem to be preponderant. These groups provide a further opportunity for learning, which end up taking place in a more dynamic manner, given that the activities are developed by the students themselves. Activities relating to theory can be conducted, such as classes, seminars, discussions on texts or presentation of clinical cases. Practical activities can also be conducted: for example, attending patients, developing scientific projects, witnessing surgical procedures, training in techniques (such as orotracheal intubation), application of dressings, and so on. It seems clear that experiencing day-to-day practice in one specific field will end up influencing the choice of specialty.^3^ Nonetheless, it is important not to allow this to interfere with the basic learning process within the undergraduate course.^4^

The regulations of an undergraduate study group should lay down what its aims are, what its members’ obligations are, how the group is to be led and what its activities should be.^5^ These rules should also define who the participants will be, through specifying which students (from which courses, years and institutions) can take part and how many places are available. Groups that are most sought after require selection procedures, usually consisting of written appraisals, given that the number of students interested in such groups often exceeds the number of places available. Currently, because of the multiprofessional nature of healthcare, it is common for undergraduate study groups to bring together students from different courses, such as medicine, nursing, physiotherapy, nutrition, speech therapy and occupational therapy.

The first undergraduate study group formed in Brazil was the Anti-Syphilis Group, which began in 1920. This as linked to the School of Medicine of the University of São Paulo and it continues to be fully active today. This clearly demonstrates the longevity that such institutions may achieve.^6^

The cost of maintaining and developing an undergraduate study group may vary greatly, depending mainly on its activities. The greater the group’s activities are, the greater the funds that it will need to raise will be. The main sources of funding for these groups are paid activities that the groups promote (such as congresses and courses), company sponsorship and funds from the medical school, among others. Some groups are even registered in notary offices, thus indicating the complexity that such student initiatives end up attaining.

Because some medical schools have significant numbers of undergraduate study groups, they already have well-established rules for guiding their creation, development and activities. Nonetheless, in most universities, their creation depends solely on the students’ motivation. This is perhaps the greatest virtue of these entities: they allow students to develop an association dedicated to learning through their own wishes.

The classes and material presented during the activities of undergraduate study groups should not be envisaged as corrections for possible failures in the formal curriculum. Rather, they should serve as the starting point for continual rediscussion and readaptation of the curriculum in the light of update requirements.^7^

Initiatives involving research are certainly the point of least controversy, since their positive impact on training is clear.^8^ Although there is still no adequate measurement for the extent to which undergraduate study groups are able to generate scientific production, it is clear that an increasingly large number of students are developing scientific initiation projects within undergraduate study group environments.

Other activities that are not always adequately explored relate to health promotion. Educational activities providing advice for the population, participation in community programs and development of health campaigns are some of the most important social activities that are within the reach of undergraduate study groups in the field of medicine.

It is undeniable that the numbers of undergraduate study groups are growing throughout Brazil. Over recent years, this development has been so large that it has given rise to the creation of state, regional and even national organizations, such as the Undergraduate Study Group Committee of the Brazilian Association of Intensive Care Medicine, the Brazilian Trauma Study Group Committee and the Brazilian Society for Undergraduate Study Groups within Clinical Medicine, among others. Nevertheless, the greatest milestone reached was certainly the creation of the Brazilian Association of Undergraduate Study Groups within Medicine (Associação Brasileira de Ligas Acadêmicas de Medicina; ABLAM), during the Eighth Brazilian Congress of Clinical Medicine in September 2006, with support from various medical entities.^9^

It is important to remember that undergraduate study groups should not be envisaged as vocational tests for future specialization and, even less, as early specialization. Nor should they be thought of as something that makes up for possible failures in the undergraduate curriculum. In this manner, their complementary nature will not be lost. Thus, undergraduate study groups represent a singular opportunity for developing extracurricular activities directed towards medical education, scientific research and health promotion that, when correctly guided, have a positive effect on the training of their participants.
